# Structured expert elicitation to inform long-term survival extrapolations using alternative parametric distributions: a case study of CAR T therapy for relapsed/ refractory multiple myeloma

**DOI:** 10.1186/s12874-022-01745-z

**Published:** 2022-10-15

**Authors:** Dieter Ayers, Shannon Cope, Kevin Towle, Ali Mojebi, Thomas Marshall, Devender Dhanda

**Affiliations:** 1 Evidence Synthesis & Decision Modeling, PRECISIONheor, 1505 West 2nd Ave #300, Vancouver, BC V6H3Y4 Canada; 2 Bristol Myers Squibb, Princeton, NJ USA

**Keywords:** Relapsed/refractory multiple myeloma, Cost-effect analyses, Expert opinion, Long-term survival models

## Abstract

**Background:**

Our aim was to extend traditional parametric models used to extrapolate survival in cost-effectiveness analyses (CEAs) by integrating individual-level patient data (IPD) from a clinical trial with estimates from experts regarding long-term survival. This was illustrated using a case study evaluating survival of patients with triple-class exposed relapsed/refractory multiple myeloma treated with the chimeric antigen receptor (CAR) T cell therapy idecabtagene vicleucel (ide-cel, bb2121) in KarMMa (a phase 2, single-arm trial).

**Methods:**

The distribution of patients expected to be alive at 3, 5, and 10 years given the observed survival from KarMMa (13.3 months of follow-up) was elicited from 6 experts using the SHeffield ELicitation Framework. Quantities of interest were elicited from each expert individually, which informed the consensus elicitation including all experts. Estimates for each time point were assumed to follow a truncated normal distribution. These distributions were incorporated into survival models, which constrained the expected survival based on standard survival distributions informed by IPD from KarMMa.

**Results:**

Models for ide-cel that combined KarMMa data with expert opinion were more consistent in terms of survival as well as mean survival at 10 years (survival point estimates under different parametric models were 29–33% at 3 years, 5–17% at 5 years, and 0–6% at 10 years) versus models with KarMMa data alone (11–39% at 3 years, 0–25% at 5 years, and 0–11% at 10 years).

**Conclusion:**

This case study demonstrates a transparent approach to integrate IPD from trials with expert opinion using traditional parametric distributions to ensure long-term survival extrapolations are clinically plausible.

**Supplementary Information:**

The online version contains supplementary material available at 10.1186/s12874-022-01745-z.

## Background

Health technology assessment (HTA) agencies commonly evaluate the cost-effectiveness of new interventions over a lifetime horizon. However, the follow-up data available in clinical trials for new interventions at the time of the evaluation are often limited. In this context, The National Institute for Health and Care Excellence (NICE) recommends fitting alternative parametric models to extrapolate survival, where model selection is informed by visual assessment, log-hazard plots, goodness-of-fit statistics, and an evaluation of plausibility of the extrapolations in terms of clinical validity [1, 2]. Recently, more flexible parametric models have been recommended for complex survival data [3], which are increasingly being proposed to assess the expected survival for new interventions, such as immunotherapies [4–6] and chimeric antigen receptor (CAR) T cell therapy [7]. As more flexible methods are used, the need to consider the plausibility of extrapolations is even more important given that these methods may yield less realistic shapes in terms of long-term hazard [3].

Jackson et al. identified the potential to integrate expert opinion regarding long-term survival estimates in 2017 [8]. However, this approach has rarely [9–11] been incorporated in cost-effectiveness analyses (CEAs), and there are very few published expert elicitation studies regarding time-to-event outcomes [12, 13]. Previous studies have elicited conditional probabilities at specific time points in terms of the proportion of patients who have experienced an event, rather than estimating survival in a more flexible survival model with multiple parameters [14, 15]. NICE has recently developed formal guidance regarding expert elicitation methods, including a reference protocol by Bojke et al. (2021) regarding how to design, elicit, and integrate feedback from experts [13]. While this highlights the growing recognition regarding the increasing role of expert elicitation as a tool to support decision-making, there are no recommendations specific to survival, which is likely to be a key model driver.

Standard practice often involves an informal consultation with experts (often 1 or more) who are presented with alternative survival extrapolations and asked to identify the most plausible model; however, this approach may be ‘misleading’ [16] and prone to bias [3]. In contrast, Cope et al. proposed to elicit estimates of long-term survival at multiple time points using the SHeffield ELicitation Framework (SHELF) [17]. This study demonstrated the feasibility of systematically integrating long-term survival estimates obtained from a formal expert elicitation study (2, 3, 4, and 5 years) with empirical clinical trial data (1.5 years of follow-up) through a case study evaluating a CAR T cell therapy for children and young adults with relapsed or refractory acute lymphoblastic leukemia [18]. This illustrated how expert opinion could be incorporated using a transparent, robust, and reproducible method to improve the understanding and clinical plausibility of long-term survival extrapolations. To our knowledge, this method has only been applied in one NICE technology appraisal for cemiplimab for treatment of metastatic or locally advanced cutaneous squamous cell carcinoma [ID1367], where the committee identified that the study was ‘clearly reported and appears to have been well-conducted’.^1^

To ensure the broader application of these methods, additional research is required to improve their ease of use. Cope et al. used fractional polynomial models assuming a binomial likelihood to combine the discrete hazards from each interval of the observed survival data from the clinical trial [17]. Rather than defining time intervals and calculating discrete hazards, using the exact event and censor times from the individual-level patient data (IPD) may improve the accuracy and align more closely with standard practice as suggested by Latimer et al. [2]. There is also a need to expand these models beyond first- and second-order fractional polynomial models to include the parametric distributions most often used for extrapolation of survival in CEAs: Weibull, Gompertz, lognormal, log-logistic, exponential, gamma, and generalized gamma [18]. Therefore, the aim of this study was to extend the traditional parametric models used to extrapolate survival for CEAs by integrating IPD from a clinical trial with estimates from experts regarding long-term survival. Here, we model survival in a Bayesian framework, using standard time-to-event data from a trial and subject the estimation to constraints determined by expert opinion as estimated through a structured elicitation exercise.

### Case study

Despite improvements in earlier lines of therapy, patients with relapsed/refractory multiple myeloma (RRMM) who have received at least 3 prior therapies, including an immunomodulatory agent, a proteasome inhibitor, and an anti-CD38 antibody (i.e. triple-class exposed [TCE]) often relapse and have limited survival [19], which has driven the development of several new therapies. Idecabtagene vicleucel (ide-cel, bb2121) reflects the first B-cell maturation antigen (BCMA)-directed CAR T cell therapy approved by the US Food and Drug Administration (FDA) for the treatment of TCE patients with RRMM who have received 4 or more prior lines of therapy [20]. Ide-cel has also been approved by the European Commission for the treatment of adult patients with TCE RRMM who have received at least 3 prior therapies, and have demonstrated disease progression on the last therapy [21]. Ide-cel demonstrated frequent, deep, and durable responses in TCE patients with RRMM based on the pivotal, phase 2, single-arm KarMMa trial (NCT03361748) [22]. However, at the time of HTA evaluations, the long-term survival estimate from KarMMa was limited to less than 24 months of follow-up (median follow-up of 13.3 months). This evidence is representative of the limited follow-up often available for many new interventions in oncology, where a novel mechanism of action makes it challenging to integrate external evidence regarding long-term survival. Therefore, this case study was used to illustrate how estimates from experts regarding long-term survival can be integrated into parametric models that would otherwise be limited to the IPD from a clinical trial.

## Methods

### Expert elicitation

**Fig. 1:**
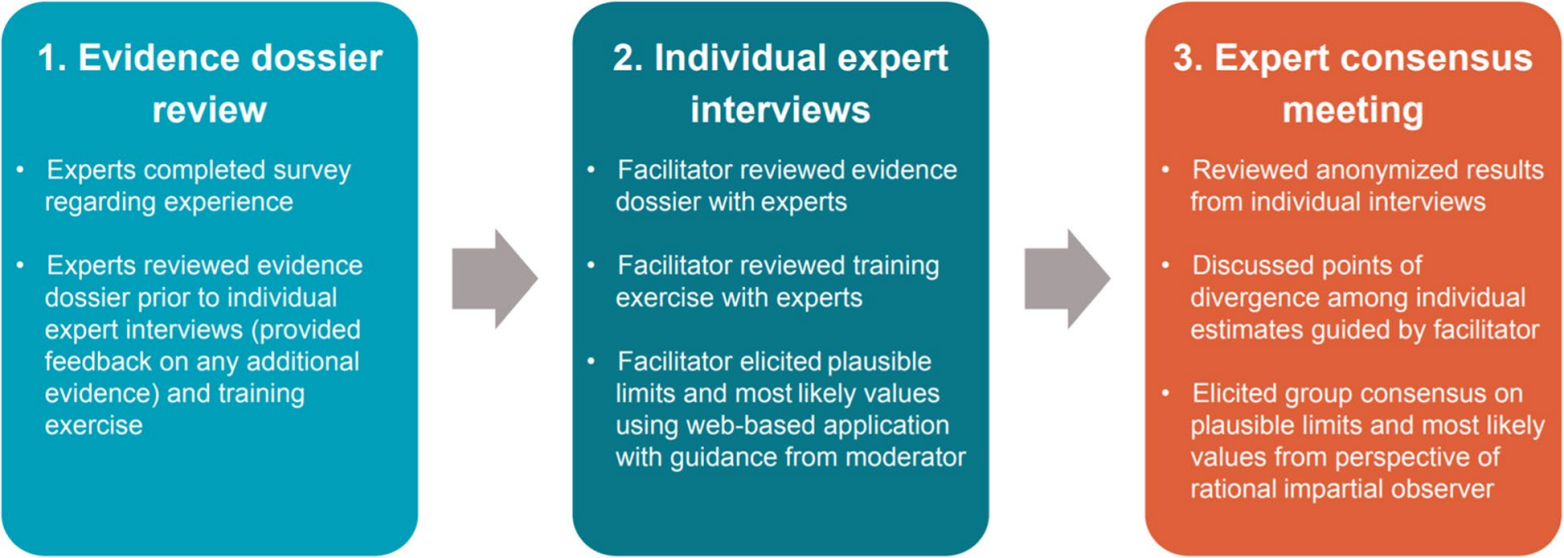
Overview of elicitation process

A prospective, qualitative, research study was performed incorporating semi-structured interviews, adapted from SHELF [23]. This study was conducted in accordance with the International Society for Pharmacoepidemiology (ISPE) Guidelines for Good Epidemiology Practices*.* We summarize the elicitation process in Fig. 1, with additional details in Additional File 1. Oncologists and hematologists with clinical experience treating TCE patients with RRMM with BCMA-directed therapy were recruited. An evidence dossier, containing relevant evidence regarding the patient population and outcomes was created to provide a common basis for expert judgments. Facilitators guided each expert through a web-based application for the elicitation of overall survival (OS), which illustrated the Kaplan–Meier (KM) curve from KarMMa, as well as the expert estimates at 3, 5, and 10 years, iteratively. At each time point, experts were first asked to estimate lower and upper plausible limits (LPLs and UPLs) and then the most likely value (MLV) for OS. During a follow-up consensus meeting, experts were presented with the (anonymized) individual estimates from each expert, and then were given the opportunity to discuss and provide rationale for divergent estimates. The experts collectively provided consensus estimates (UPL, LPL, and MLV) for OS at each time point from the perspective of a ‘rationale impartial observer’.

### Model parameterization

Parametric survival analysis defines the survival function, S(t) as the probability of surviving beyond a given time *t*. This is characterized as the complement of a cumulative distribution function for any arbitrary survival distribution:

1 $$S\left(t,{\varvec{\theta}}\right)=P\left(T>t|{\varvec{\theta}}\right)=1-\mathrm{F}\left(t,{\varvec{\theta}}\right)$$

### Integration of expert opinion

Expert consensus MLVs and plausible limits for OS were used to define a distribution of expected survival at each time point. Conceptually, the MLV corresponds to the mode, which is operationalized as the mean in the context of a normal distribution. A truncated normal distribution was used to define the consensus distribution at each time point to align with the following constraints: 1) survival be bounded by 0 and 1; and 2) plausible limits and MLVs can coincide (i.e. survival at 10 years is expected to be 0, thus the MLV and the lower limit are both 0). To accommodate both constraints, the distribution of plausible survival probabilities, Y, at each elicitation time point *j* was therefore defined as:

2 $${\mathrm{Y}}_{j} \sim \mathrm{N }\left({\upmu }_{j},{\upsigma }_{j}^{2}\right)\mathrm{I}\left({0<\mathrm{Y}}_{j}<1\right)$$

where ${\upmu }_{j}$ is the mean (and mode) of the distribution before truncation, and the variance, ${\upsigma }_{j}^{2}$, is based on the width of the interval provided by the UPL and LPL for the expert estimates, which were assumed to reflect the 99^th^ and 1^st^ percentiles. For each survival estimate provided by experts, ${\upmu }_{j}$ is related to the survival distribution by setting ${\upmu }_{j}$ to the expected survival based on the survival model:

3 $${\upmu }_{j}=S\left({t}_{Ej},{\varvec{\theta}}\right)$$

Tying the survival function S(t) to the mean of the experts’ probability distribution of Y effectively added constraints to the possible values of the parameters. The strength of these constraints was determined by the confidence of the experts, as measured by the variance of the distribution.

For the i = 1,…,N subjects in the trial, T_i_ is the time of event or censoring, and δ_i_ is an indicator (δ_i_ = 1 if the event is observed and δ_i_ = 0 for censored observations). The survival distribution is defined with a density f(t_i_|**θ**) and a cumulative distribution function F(t_i_|**θ**) with parameters **θ**. The estimates of the experts’ MLV, Y_j_, was obtained at M time points {t_E,1_,…t_E,M_}, and have a density of d(Y_j_|θ), the truncated normal distribution defined by Eqs. (2) and (3).

The full Bayesian model specifies the following joint probability:

4 $$\mathrm{Pr}\left({\varvec{\theta}} |T,\delta ,Y\right)\propto \mathrm{Pr}\left(T,\delta ,Y|{\varvec{\theta}} \right)\mathrm{Pr}\left({\varvec{\theta}} \right)=\left(\prod_{i=1}^{N}{f({T}_{i}|{\varvec{\theta}} )}^{{\delta }_{i}}{(1-F({T}_{i}|{\varvec{\theta}} ))}^{(1-{\delta }_{i})}\right)\left(\prod_{j=1}^{M}d({Y}_{j}|{\varvec{\theta}} )\right)\mathrm{p}({\varvec{\theta}} )$$

### Analysis

Separate parametric models were evaluated based on: 1) observed OS from KarMMa (without expert opinion); and 2) observed OS from KarMMa in combination with the expert consensus estimates of OS at 3, 5, and 10 years. The following parametric models were evaluated: Weibull, Gompertz, lognormal, log-logistic, exponential, gamma, and generalized gamma. Specific parameterizations are shown in Supplementary Table 2, Additional File 2. Individuals who did not experience death were censored as outlined by Qi et al. [24]. Analyses were performed in the Bayesian framework with approximately non-informative prior distributions, assuming a gamma distribution (1.0 × 10^–3^, 1.0 × 10^–3^) for parameters that were strictly positive, and a Normal (0,τ = 0.001) distribution for real-valued parameters taking values on the real number line (Additional File 3 presents the analysis and JAGS code).

The R SHELF package was used to obtain the mean and variance of the probability distributions estimated by experts from the consensus meeting. The parameters were estimated using a Markov Chain Monte Carlo (MCMC) method as implemented in Just Another Gibbs Sampler (JAGS) (version 4.3.0) (https://sourceforge.net/projects/mcmc-jags/files/) and R (version 4.0.4) (http://www.r-project.org) software packages. For distributions with built-in functions in JAGS, the observed event times were used to estimate the parameters of the selected distribution. In the absence of simple specifications for log-logistic and Gompertz distributions in JAGS, we used the zeros trick to specify the likelihoods directly. A first series of 20,000 iterations from the JAGS sampler was discarded as ‘burn-in’ and the inferences were based on 50,000 additional iterations using 2 chains. Convergence of the chains were confirmed by the Gelman-Rubin statistic. Deviance information criterion (DIC) was used to compare to the goodness of fit to the data (with or without expert information). Results were illustrated in terms of survival curves with 95% credible intervals (CrIs) and the area under the curves (up to 10 years).

## Results

### Expert elicitation

**Fig. 2:**
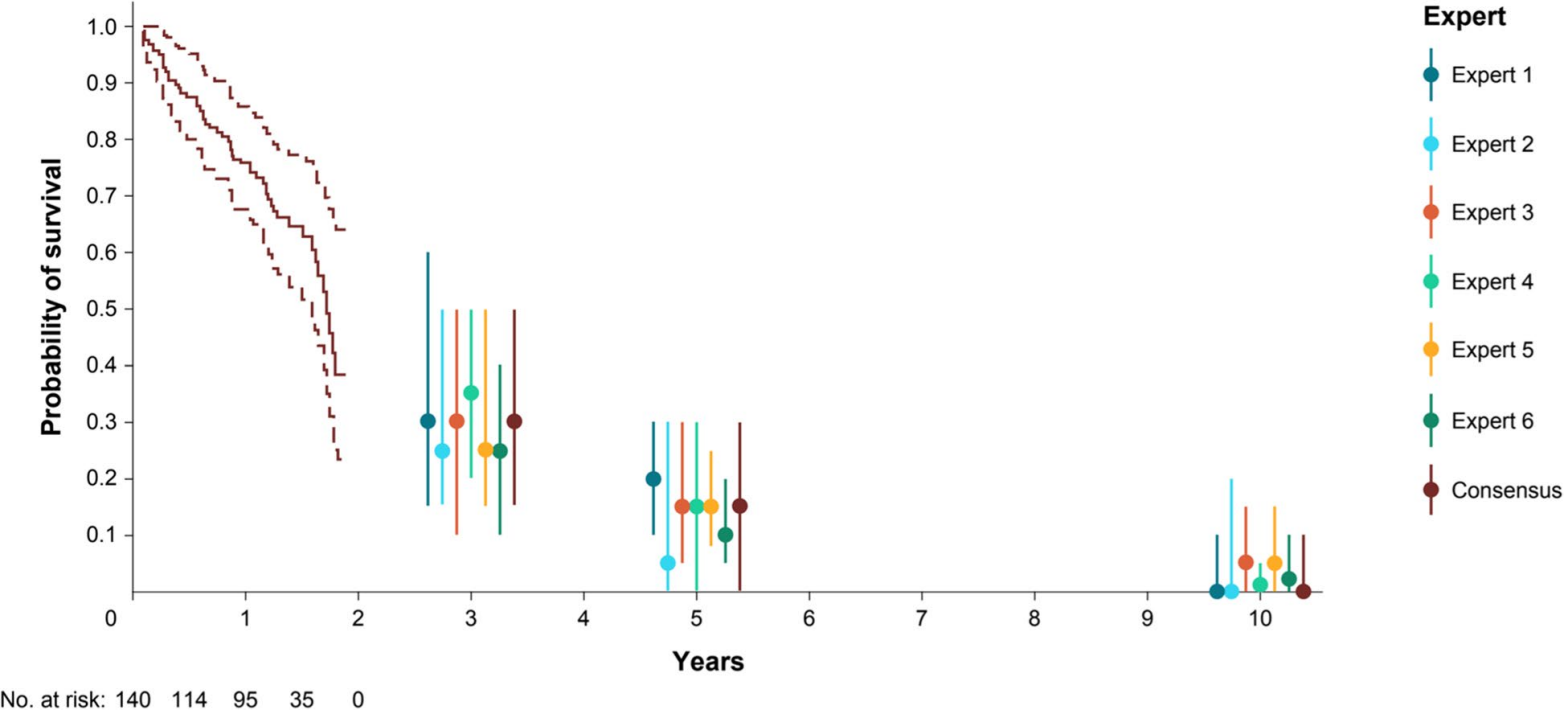
Expert-specific and consensus survival estimates at each time point of interest based on KarMMa for patients treated with ide-cel. Observed data includes OS curve (solid line) and associated 99% CI (dashed lines). Dots represent most likely values, and vertical bars show the plausible range. Abbreviations: CI, confidence interval; ide-cel, idecabtagene vicleucel; OS, overall survival

Experts (*n* = 6) had extensive experience treating the population of interest (Supplementary Table 1, Additional File 1). Survival estimates from experts given KarMMa patients treated with ide-cel for the MLV ranged from 25 to 35% at 3 years, 5 to 20% at 5 years, and 0 to 5% at 10 years (Fig. 2). There was more variation across experts at earlier, as compared to later, time points, although estimates did not vary substantially overall. Survival tended to decline gradually from 3 to 10 years in most cases, whereas Expert 1 suggested a sharper reduction at 10 years. Some experts were more optimistic (Expert 1) as compared to others (Experts 2 and 6) and some were more certain (Experts 4 and 6) as compared to others.

**Fig. 3:**
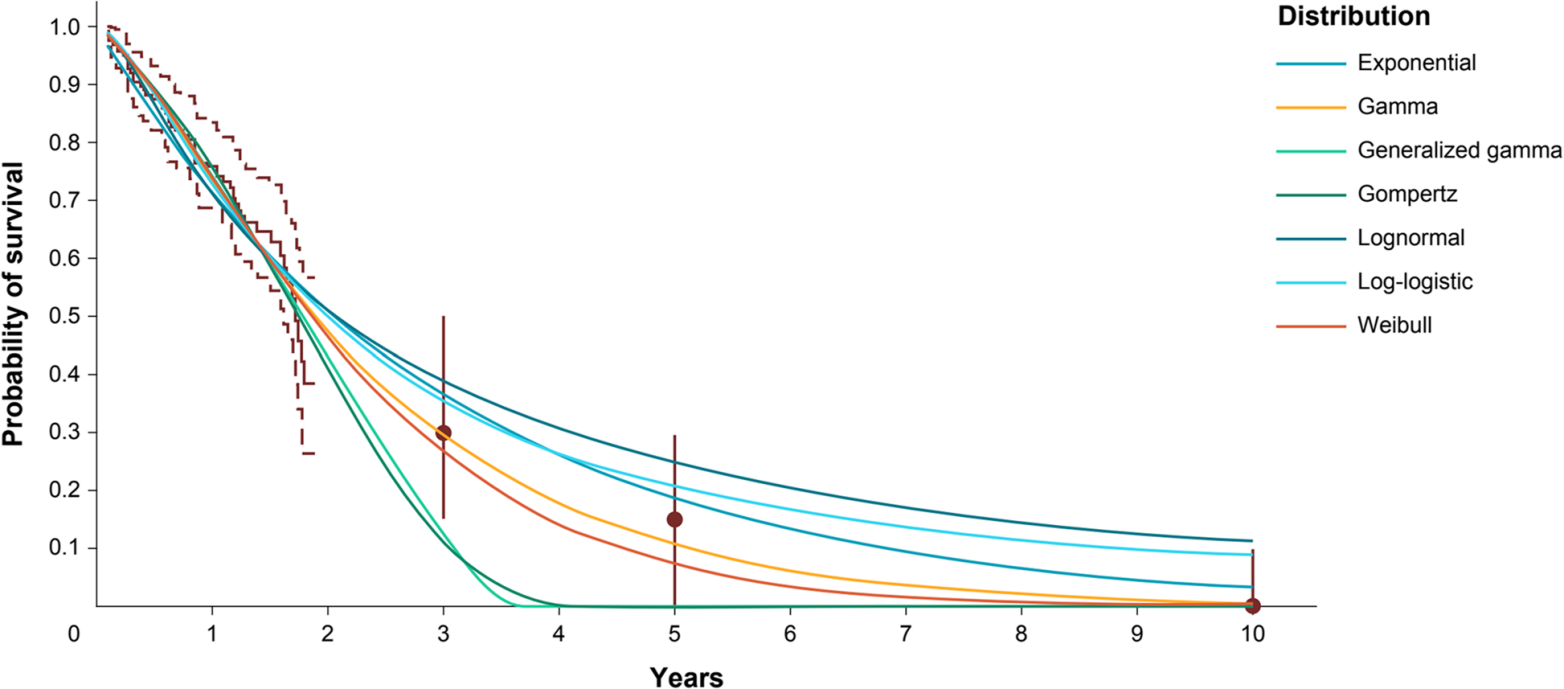
Long-term survival estimates based on observed KarMMa data (without expert opinion). Observed data includes OS curve (solid line) and associated 99% CI (dashed lines). Dots represent consensus most likely values, and vertical bars show the plausible range. Abbreviations: CI, confidence interval; OS, overall survival

Within the range of the observed KarMMa data (median follow-up of 11.3 months; maximum 22.6 months), the alternative parametric models were reasonably similar. At extrapolated time points, however, the models diverged. Point estimates ranged from 11 to 39% at 3 years, 0 to 25% at 5 years, and 0 to 11% at 10 years (Fig. 3).

**Fig. 4:**
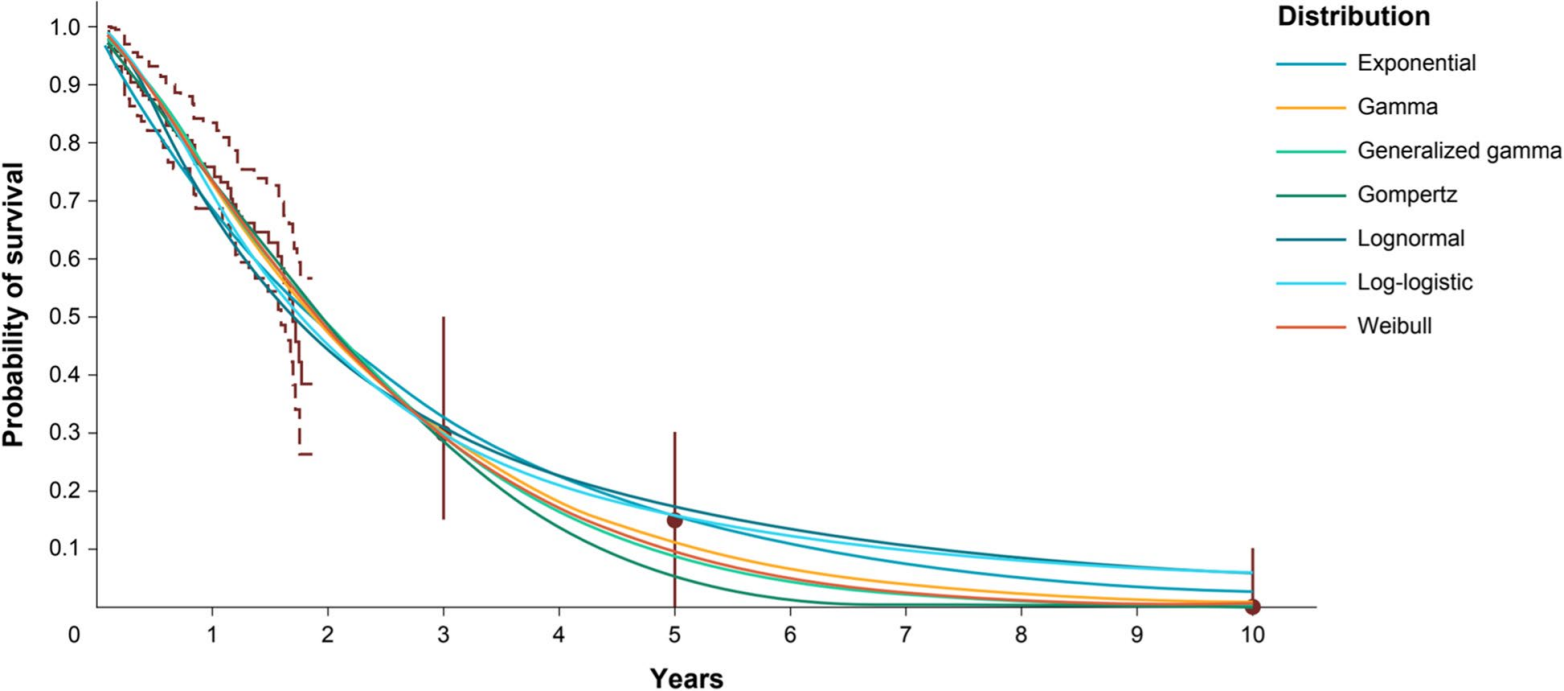
Long-term survival estimates based on observed KarMMa data and consensus expert opinion. Observed data includes OS curve (solid line) and associated 99% CI (dashed lines). Dots represent consensus most likely values, and vertical bars show the plausible range. Abbreviations: CI, confidence interval; OS, overall survival

**Fig. 5:**
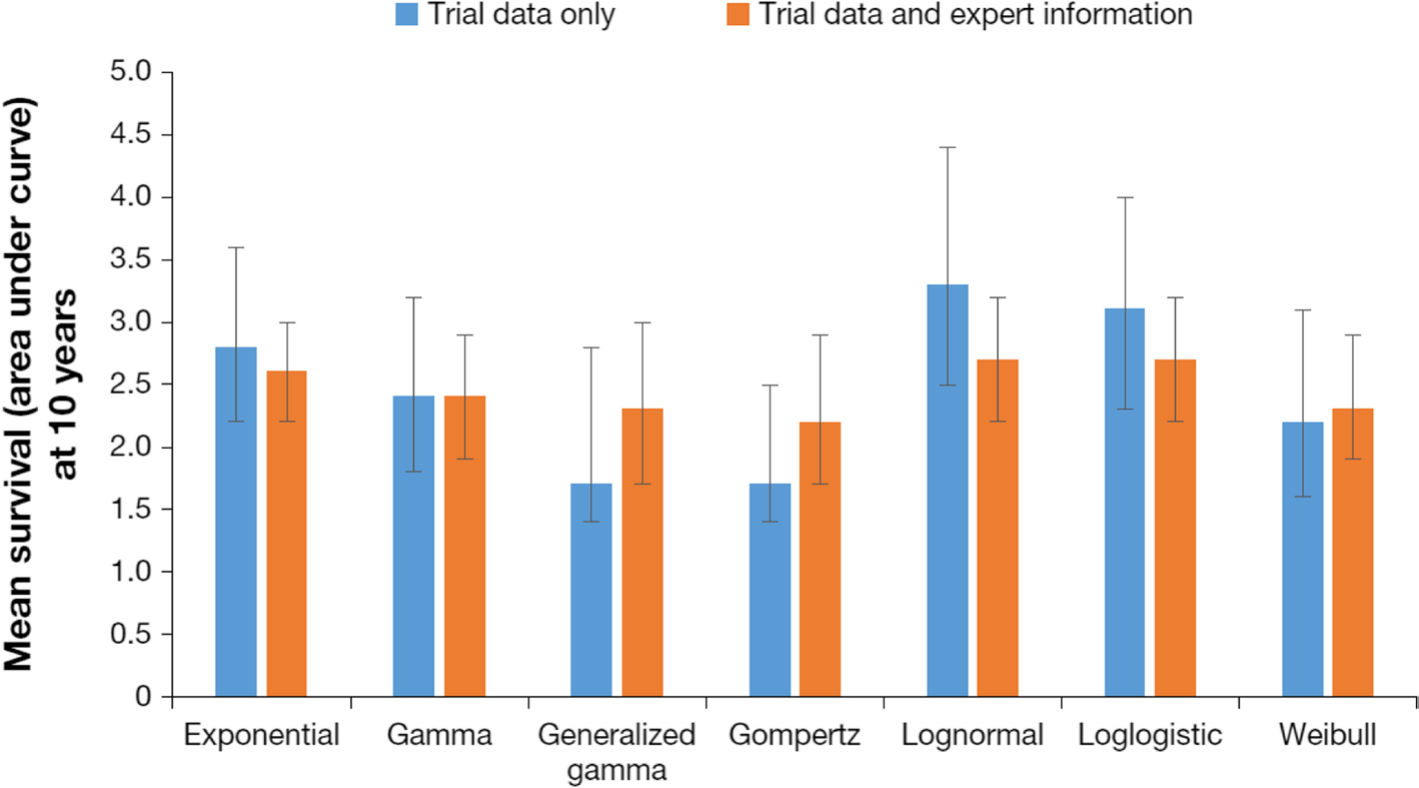
Mean survival (AUC) at 10 years for patients treated with ide-cel based on KarMMa using trial data only or combining trial data with expert information. Abbreviations: AUC, area under the curve; ide-cel; idecabtagene vicleucel

**Fig. 6:**
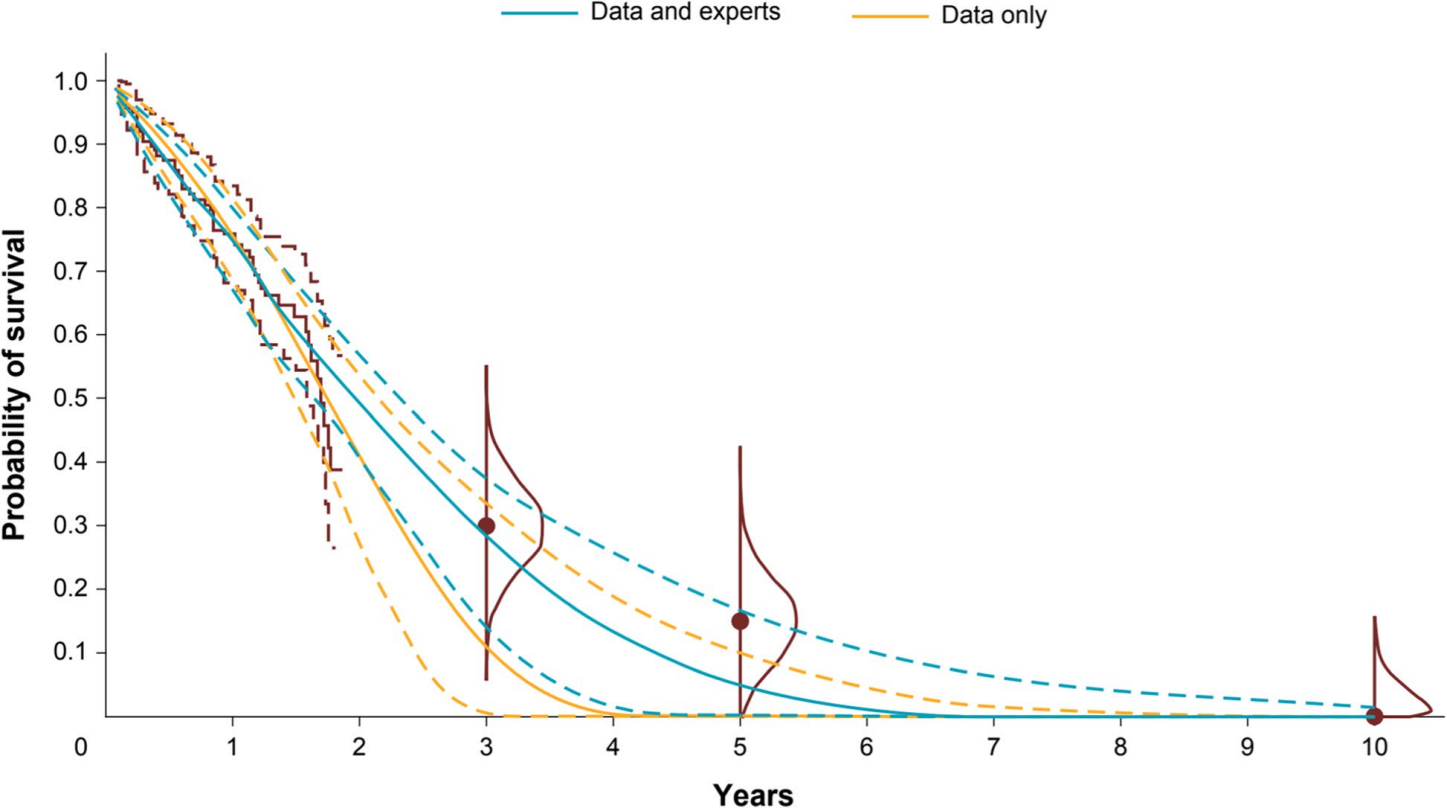
Comparison of Gompertz models based on KarMMa data alone and KarMMa data and consensus expert opinion. Dotted blue and yellow lines reflect the 95% credible intervals. Observed data includes OS curve (solid line) and associated 99% CI (dashed lines). Dots represent consensus MLV, and densities show the truncated normal distributions based on the expert-defined plausible range. Abbreviations: CI, confidence interval; MLV, most likely value; OS, overall survival

When the models combined the observed OS data from KarMMa with expert opinion, the expert information led to more consistent estimates across the parametric models as compared to the models without expert information (Fig. 4). OS point estimates ranged from 29 to 33% at 3 years, 5 to 17% at 5 years, and 0 to 6% at 10 years. Supplementary Figs. 3 and 4, Additional File 4, present 95% CrIs for the survival extrapolations in Figs. 3 and 4, respectively. Figure 5 illustrates the mean survival at 10 years, which reinforces how the point estimates across parametric distributions align more closely following integration of expert information and estimates of uncertainty were reduced. Model selection can be based on the DIC values presented in Table 1. Based on the trial data alone, the Gompertz model resulted in the lowest DIC, although there was only a 5-point difference across the models. When expert opinion was considered along with the trial data, the Weibull and Generalized Gamma models had the lowest DICs and the estimates differed more (14 points), helping to differentiate which models aligned most closely with expert opinion. This can be used as a rationale to support the selected models without expert information, or alternatively the estimates that integrate expert opinion can be used directly. The Gompertz and Weibull models emphasize how the effect of expert opinion can differ depending on the underlying distribution. Beyond the range of the trial data, the Gompertz predicts very low survival when using observed OS data alone. Notably, the upper bound of the model without expert opinion was near the consensus MLV at 3 years and excludes it at 5 years, demonstrating that this model was not consistent with expert opinion. Consequently, incorporating expert opinion for this model led to a substantial increase in survival at the 3- and 5-year time points. Using the Weibull distribution resulted in similar changes, although the model without expert information was more consistent with expert estimates and therefore led to less change when expert information was incorporated. For these 2 distributions, the differences in prediction and CIs between models with observed OS data alone (without expert opinion) and models with observed OS and expert opinion are shown in Fig. 6.

**Table 1:** Model fit statistics for all models

	Without experts			With experts		
	Deviance	Penalty	DIC	Deviance	Penalty	DIC
Exponential	476.61	0.93	477.55	465.23	1.10	466.33
Weibull	472.74	2.09	474.83	460.51	2.14	462.65
Lognormal	476.04	2.08	478.12	468.18	1.88	470.06
Log-logistic	474.82	2.05	476.86	475.01	1.90	476.91
Gamma	473.04	1.91	474.94	460.66	2.04	462.71
Generalized gamma	471.54	2.69	474.23	463.39	3.33	466.72
Gompertz	470.99	1.90	472.89	472.68	1.74	474.42

## Discussion

There is increasing interest in methodology to accurately extrapolate estimates of survival beyond the clinical trial follow-up period [3, 11, 18, 25]. Important differences in the mean incremental cost-effectiveness ratios and their uncertainty have been identified using traditional parametric models to extrapolate survival in cases where hazards were constant, increasing, decreasing, or unimodal [26]. In another case study, including more flexible models to extrapolate a single-arm clinical trial led to estimates of expected survival (i.e. area under the curve) ranging from 1.19 (Weibull) to 2.11 (log-logistic) to 3.31 (splines) to 11.22 (Weibull mixture model) years [4]. Given the impact of this structural uncertainty, current interest focuses on incorporating information external to clinical trials to limit the extrapolated survival to values that are reasonable, based on this additional information.

We have formally elicited the opinions of subject-matter experts and incorporated those beliefs into parametric survival models. Expert opinion has the benefit of being relevant to the population of interest, as the experts take this into account during the elicitation, which may not be the case with other external sources. Previously, Cope et al. [17] demonstrated the feasibility of systematically integrating long-term survival estimates obtained from a formal expert elicitation study with empirical clinical trial data. This provides a transparent, robust, and reproducible method to incorporate expert opinion into the model selection process [17]. We have extended this work to integrate the exact individual event and censor times based on the IPD using parametric models used most often for CEAs, rather than fitting fractional polynomials to the discrete hazards. We present the JAGS code for these models with and without expert information to encourage others to use or adapt these models for future HTAs that require CEAs. Including expert opinion does not fundamentally alter the structure of the model, which makes it easy to incorporate into a CEA model. The survival distributions are defined primarily by the data, which are modified by the expert information, while still retaining the general properties of the survival distributions. This allows for a single model that is not subject to additional subjective modeling choices such as knot placements (as required by splines), cut points, or classification into disjoint sets of patients (as in mixture models).

Using data from the KarMMa trial helps to illustrate these models and highlights the increased consistency across the models once expert opinion has been integrated, which aims to improve the plausibility of the extrapolations from a clinical perspective. As seen with the Gompertz model, the inclusion of the expert data removes the influence of the drop in survival at the end of the trial. The lognormal distribution is characterized by hazards that decrease in the tail, which often leads to an unrealistic plateau in survival when extrapolated. Therefore, expert opinion pulled this survival down into a more plausible range. This is particularly notable in our case study of heavily pretreated TCE patients with RRMM, who have poor survival outcomes.

As the experts do not change the underlying parametric model, it is possible that expert opinion is not consistent with a particular model, and that the model is not sufficiently flexible to incorporate the expert information. This was the case for the lognormal distribution, where adding expert information came at the expense of lowering the curve within the range of the trial data. Therefore, the notable differences between the lognormal models with and without expert information can be used as evidence that this distribution is not well suited to the data. However, the other distributions could be adjusted by the expert information and produced plausible long-term survival predictions that fit well to the 3- and 10-year expert estimates, whereas the 5-year survival estimates were slightly lower than the expert information, but still within the plausible ranges. Therefore, the influence of the expert information may depend on the assumed survival distribution. It may be of interest to extend our approach to more flexible models, such as cubic splines, fractional polynomials, or mixture models, as described in recent guidance by the NICE Decision Support Unit, where constraints may be increasingly important to ensure plausible estimates [3].

Our approach is similar to Guyot et al., who also constrained the parameter estimates based on conditional survival from either experts or observational data [11]. However, we address the challenge of integrating sources of evidence where we have the survival estimates (at multiple time points) rather than the number of patients at risk and with an event. Guyot et al. [11] also constrained the treatment effects in terms of the hazard ratio, forcing it to be one at a specific time point with a certain degree of uncertainty. Future research could extend our models to include treatment effects for a new intervention versus standard of care to integrate evidence from randomized controlled trials (or more broadly to indirect comparisons and network meta-analysis models versus multiple comparators of interest). In this context, it may be interesting to explore the impact of asking experts regarding survival estimates for each treatment arm separately, versus directly asking about constraints on the treatment effect(s) (either at specific time points or asking when the treatment effect would be expected to return to one).

Beyond imposing a functional relationship in the parameters of the survival distribution there are alternative methods to share information as outlined by Nikolaidis et al. [27]. External evidence can be integrated as prior information, which is typically the approach to integrate expert opinion. In the context of survival outcomes, Soikkeli et al. used mature historical trial data as prior information to inform the shape parameter of a comparator arm of a pivotal trial [28]. In our study, experts did not provide direct information on the parameters of survival distributions, but rather were asked about survival at specific times in the tail of the distribution, which complicated the integration using priors. Since the KM plot of survival from the trial was presented to experts to inform their long-term estimates, the trial itself could have been used to inform the priors for the survival parameters, while modeling the effect of expert information; however, we preferred a functional model given the dependency between the KM and the expert estimates. This approach also allows for the integration of other sources as priors, such as evidence from phase 1 trials to inform longer-term follow-up, whereas historical control evidence used by Soikkeli et al. [28] is less likely to be available regarding new interventions. Mixture priors (Efthimiou et al. [29]) or power priors (Rietbergen et al. [30]) could be used to down-weight evidence depending on differences between the phase 1 and the pivotal trial. However, it may be necessary to consider how experts may have incorporated this earlier trial information into their estimates. 

Rather than integrating external information as functional relationships or using priors, it may be possible to develop a multilevel model to integrate different sources of evidence similar to how Schmitz et al. combined information from different study designs [31]. This may have the advantage of being able to explicitly control the relative weight of the estimates from the trial versus the experts. Also, such a model might allow for the combination of estimates from each individual expert as well as estimates of between-expert variation (given sufficiently large number of experts). Depending on whether estimates from experts alone provides sufficient information to provide stable estimates, future research could evaluate whether this would be feasible in the context of piecewise or spline models that are interval specific.

One potential limitation of the current model is that the uncertainty in expert estimates is not directly linked to the sample size and event rate of the clinical trial in the model. Therefore, while a larger study will carry more weight than a smaller study relative to the same expert estimates, it is not clear how the uncertainty in expert estimates relates to the sample size of the trial. Future research could explore this by evaluating alternative scenarios in which the expert elicitation varies sample size, or by asking experts to directly estimate the number of patients at risk at the end of the trial who would be expected to have died. Adding a parameter to up- or down-weight expert estimates in the model in relation to expert uncertainty may provide a straightforward approach to explore the relative weight of experts versus the trial. This may help to mitigate potential limitations regarding the case study, such as potential bias in the elicitation process, recruitment of experts, and number of experts (Additional File 1).

Finally, the current analysis used truncated normal distributions to characterize the expert distributions at each time point, which aligned reasonably well with the estimates provided by experts. Using a beta distribution may fit more naturally with the estimated survival probabilities provided by experts. However, at 10 years, both the MLV and the LPLs were 0, which was not feasible to incorporate with the beta distribution. As a possible extension, a multivariate distribution could be used to account for the correlation between estimates at different time points. Additional research regarding the optimal time points for the elicitation of the survival estimates would be helpful. Conditional probabilities at particular time points were selected as the quantity of interest to ensure it was straightforward for experts to understand and elicit; however, alternative quantities of interest for time-to-event outcomes could be explored. Finally, it is unclear whether the experts’ uncertainty is truly equivalent to the sampling distribution of the MLV. A different set of experts may provide a different measure of variability, which could affect the model fits. The use of the consensus values should mitigate this; however, this still assumes that the distributions derived from expert opinion are representative of the variability of S(t).

Our case study in TCE RRMM provides a representative example where expert opinion regarding long-term survival adds information for a CAR T cell therapy with a novel mechanism of action, where there is limited evidence beyond the available follow-up from the phase 2 study (*N* = 140). Given the unmet need in these heavily pretreated patients, the approval of this new therapy provides an important new treatment option, which will also be explored in earlier lines of therapy (NCT03651128). As long-term survival estimates become more favorable, the importance of getting the tail right reinforces the role of formal integration of expert opinion.

## Conclusions

Overall, this study demonstrates a structured and transparent approach to integrate IPD from a clinical trial with expert opinion using traditional parametric models to ensure long-term survival extrapolations are plausible.Our methodology improves upon current model selection methodology to directly integrate expert opinion, which may improve the process for CEAs and decision-making for HTA. This will be increasingly important to constrain more flexible parametric models as recommended in most recent NICE Decision Support Unit guidance.

## Supplementary Information

**Additional file 1.** Details of the elicitation methods. Table 1. Summarized results from clinical experience background survey. Fig. 1. Example of web-based application for expert elicitation exercise (simulated data) prior to estimations. Fig. 2. Example of web-based application for expert elicitation exercise (simulated data) after plotting estimates.

**Additional file 2.** Parameterizations of alternative models. Table 2. Parameterizations of survival distributions employed in analyses.

**Additional file 3.** Analysis R code for survival analysis with and without expert opinion and corresponding JAGS code.

**Additional file 4: **Fig. 3. Long-term survival estimates and 95% CrIs based on observed KarMMa data (without expert opinion). Fig. 4. Long-term survival estimates and 95% CrIs based on observed KarMMa data and consensus expert opinion.

## Data Availability

Datasets generated and analyzed for this study can be made available upon request as per the Bristol Myers Squibb’s policy on data sharing found at https://www.bms.com/researchers-and-partners/independent-research/data-sharing-request-process.html.

## Abbreviations

AUC: Area under the curve
BCMA: B-cell maturation antigen
CAR: Chimeric antigen receptor
CEA: Cost-effectiveness analysis
CI: Confidence interval
CrI: Credible interval
DIC: Deviance information criterion
FDA: US Food and Drug Administration
HTA: Health technology assessment
ide-cel: Idecabtagene vicleucel
IPD: Individual-level patient data
JAGS: Just Another Gibbs Sampler
KM: Kaplan-Meir
LPL: Lower plausible limit
MCMC: Markov Chain Monte Carlo
MLV: Most likely value
NICE: National Institute for Health and Care Excellence
OS: Overall survival
PFS: Progression-free survival
RRMM: Relapsed/refractory multiple myeloma
SHELF: SHeffield ELicitation Framework
TCE: Triple-class exposed
UPL: Upper plausible limit

## Footnotes

1 https://www.nice.org.uk/guidance/ta592/documents/committee-papers
