# Supplementary material for: Structured expert elicitation to inform long-term survival extrapolations using alternative parametric distributions: a case study of CAR T therapy for relapsed/ refractory multiple myeloma
Source: BMC Med Res Methodol. 2022 Oct 15;22:272. doi: 10.1186/s12874-022-01745-z (PMC9569052; doi:10.1186/s12874-022-01745-z)
Supplement: Supplementary file 1 — Additional file 1. Details of the elicitation methods. Table 1. Summarized results from clinical experience background survey. Fig. 1. Example of web-based application for expert elicitation exercise (simulated data) prior to estimations. Fig. 2. Example of web-based application for expert elicitation exercise (simulated data) after plotting estimates. [file 12874_2022_1745_MOESM1_ESM.docx]

# Additional File 1. Details of the elicitation methods

This study was conducted in accordance with the International Society for Pharmacoepidemiology (ISPE) Guidelines for Good Pharmacoepidemiology Practice (GPP). The expert recruitment and study protocol were reviewed and approved by Advarra (Columbia, MD, USA), an Independent Review Board (IRB) that is fully accredited by the Association for the Accreditation of Human Research Protection Programs (AAHRPP) and registered with the Food and Drug Administration (FDA) and the Office for Human Research Protections (OHRP) within the United States Department of Health and Human Services (HHS).

## Expert selection

Experts were required to: 1) be board certified in oncology and/or hematology; 2) practiced oncology for at least 5 years; 3) specialized in multiple myeloma; 4) had treatment experience with triple-class exposed (TCE) patients with relapsed/refractory multiple myeloma (RRMM), including treatment experience with B-cell maturation antigen (BCMA)-targeted therapy; and 5) were English-language proficient. Experts represented various geographic regions, including the United States, Canada, Germany, the Netherlands, Germany, and Spain. Experts had an average of 25 years of experience (range: 17–30 years), including specialists in medical oncology (*n* = 3), internal medicine (*n* = 4), and/or hematology (*n* = 3) with extensive experience with TCE patients with RRMM, BCMA-directed therapy, and idecabtagene vicleucel (ide-cel) (treated between 1 and 3 patients [*n* = 2 experts], 4 and 9 patients [*n* = 3 experts], and 10 and 19 patients [*n* = 1 expert] over the past year) (Supplementary Table 1, Additional File 1). Although experts were required to have experience with BCMA-directed therapy, the depth of this experience was not likely to exceed 2 years, given that the first BCMA-targeted antibody–drug conjugate, belantamab mafodotin, was approved in 2020 by the FDA.

**Table 1** Summarized results from clinical experience background survey

| Expert characteristics | Experts (N=6) |
| --- | --- |
| **% of time spent providing direct patient care, n (%)** | |
| 25–49% | 1 (17) |
| 50–74% | 2 (33) |
| 75–100% | 3 (50) |
| **Number of years spent practicing oncology** | |
| 11–15 years | 1 (17) |
| 16–20 years | 3 (50) |
| >20 years | 2 (33) |
| **Number of MM patients treated per month (all stages), n (%)** | |
| 51–100 patients | 3 (50) |
| >100 patients | 3 (50) |
| **Number of RRMM patients treated per month (all stages), n (%)** | |
| 20–49 patients | 4 (67) |
| >50 patients | 2 (33) |
| **Number of triple-class exposed RRMM patients treated per month (all stages), n (%)** | |
| 1–3 patients | 0 (0) |
| 4–9 patients | 1 (17) |
| 10–19 patients | 1 (17) |
| 20–49 patients | 4 (67) |
| >50 patients | 0 (0) |
| **Number of patients treated with BCMA-targeting agents over past year, n (%)** | |
| 1–3 patients | 0 (0) |
| 4–9 patients | 1 (17) |
| 10–19 patients | 3 (50) |
| 20–49 patients | 1 (17) |
| >50 patients | 1 (17) |
| **Number of patients treated with ide-cel over past year, n (%)** | |
| 1–3 patients | 2 (33) |
| 4–9 patients | 3 (50) |
| 10–19 patients | 1 (17) |
| 20–49 patients | 0 (0) |
| >50 patients | 0 (0) |

Abbreviations: BCMA, B-cell maturation antigen; ide-cel, idecabtagene vicleucel; MM, multiple myeloma; RRMM, relapsed/refractory multiple myeloma

## Evidence dossier

An evidence dossier was developed to summarize KarMMa and provide a common basis for expert opinions. Experts were asked to review the evidence dossier prior to the individual interviews to identify whether any information was missing or should be revised. This review was requested to ensure that the evidence was comprehensive and that it could be revised in advance of the elicitation exercise.

The quantities of interest were defined as:

1. The expected proportion of patients in the study that are alive at 3 years, given the observed Kaplan-Meier (KM) overall survival (OS) curve (based on the available follow-up data).
2. The expected proportion of patients in the study that are alive at 5 years, given the estimated proportion alive at 3 years.
3. The expected proportion of patients in the study that are alive at 10 years, given the estimated proportion alive at the earlier time points.

The number of time points were designed to ensure there was sufficient information to derive the shape of the long-term survival, while minimizing the number of estimates experts needed to provide. The selected time points were designed to align with the available follow-up from the trials, experience from a previous elicitation in relapsed/refractory acute lymphoblastic leukemia [17], and information regarding long-term survival from heavily pretreated RRMM patients estimated in context of previous health technology assessment (HTA) evaluations. However, TCE patients represent a more heavily pretreated population with poorer outcomes in comparison to the previous RRMM populations. Therefore, inclusion of earlier time points, such as 8 years, may have provided additional information on the shape of survival curves and may have affected estimation at longer timeframes. Beyond the number of time points, the estimates in relation to each time point was restricted to the plausible limits and most likely value (MLV) rather than including additional information regarding the distribution (i.e. tertiles, quartiles etc.). We limited to 3 time points with the MLV and plausible limits to avoid overcomplicating the exercise and potentially jeopardizing the quality of the estimates by introducing estimation fatigue.

## Individual expert interviews (individual-level elicitation)

A web-based application was used to plot the KarMMa observed KM OS curve and each estimate from the expert in real time. An example of the application is shown in Supplementary Figs. 1 and 2, Additional File 1, where hypothetical predictions are shown for an example data set. A plausible limit was defined as a value the expert judged to be extremely unlikely that the true value be above (upper plausible limit [UPL]) or below (lower plausible limit [LPL]), consistent with SHELF methodology. The 99% confidence intervals (CIs) from observed KM OS curves were plotted and explained to be analogous to the plausible limits.

Before eliciting the survival estimates at specific time points, experts were asked to estimate the time when they expected all patients to have died in the relevant study (i.e. when survival equaled 0%) in order help experts ‘anchor’ themselves and consider the potential shape of long-term survival. To understand the rationale for their estimates, each expert was asked to identify sources of information that informed their opinions and provide any justification.

During individual interviews, the experts reported that they considered the following factors in their estimation process: the observed study data, including the number of patients at risk, the underlying study populations, the recruitment/study period, the availability of interventions, and their own clinical experience.

**Fig. 1** Example of web-based application for expert elicitation exercise (simulated data) prior to estimations


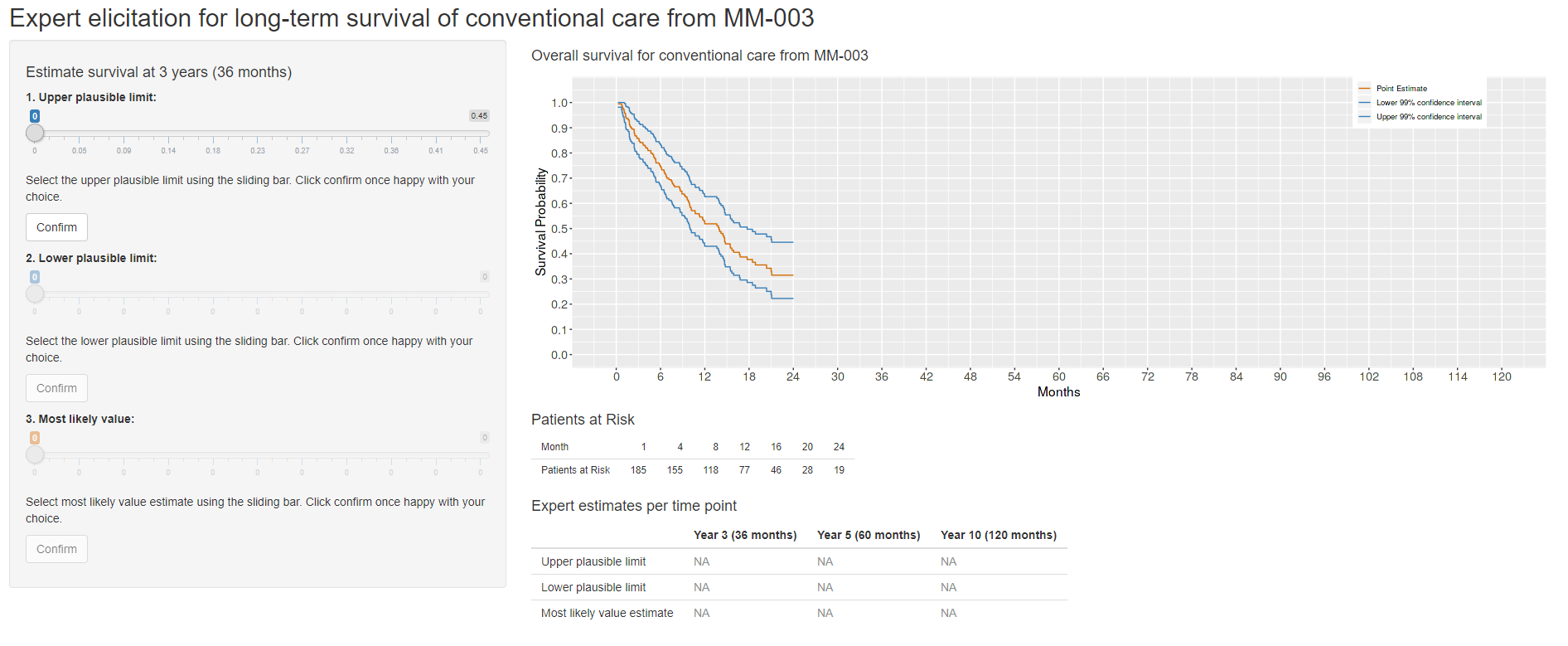


The orange line represents the observed OS Kaplan-Meier curve and the blue lines represent the 99% CI for the OS curve. Abbreviations: CI, confidence interval; OS, overall survival

**Fig. 2** Example of web-based application for expert elicitation exercise (simulated data) after plotting estimates


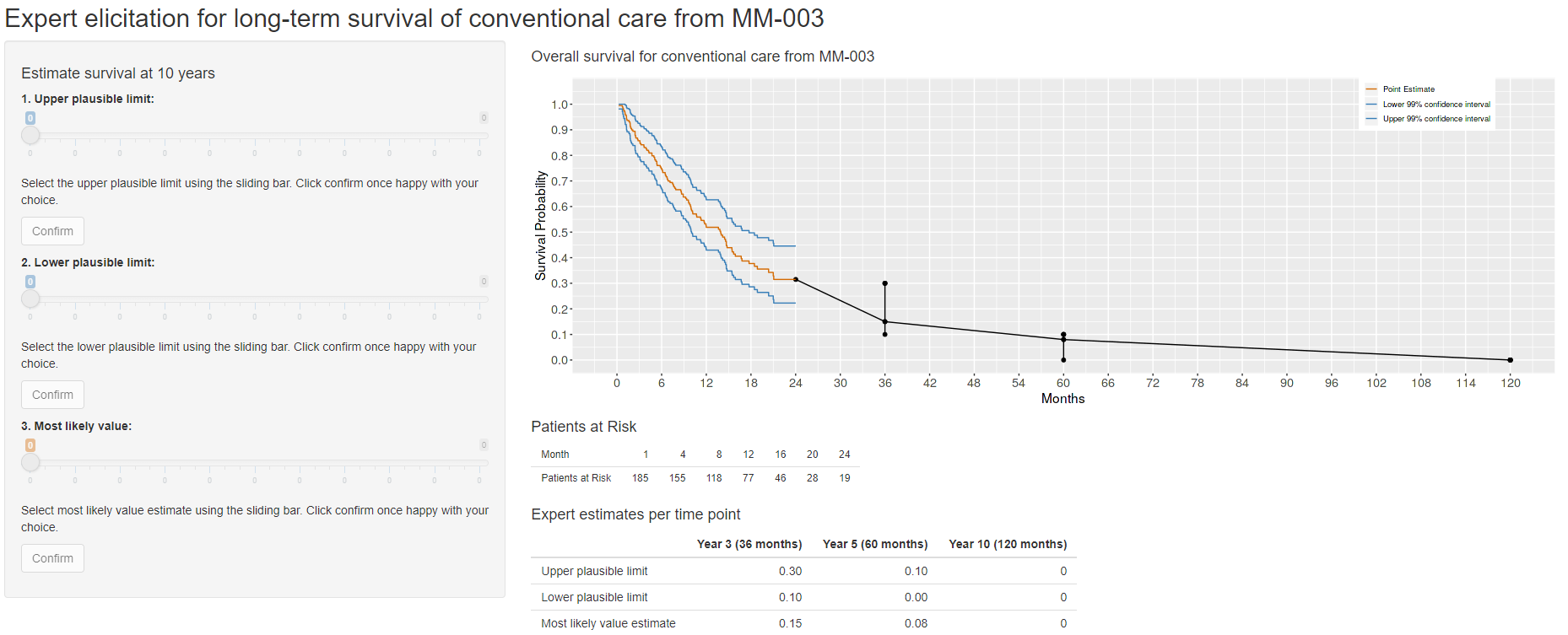

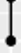

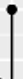


The orange line represents the observed OS Kaplan-Meier curve, and the blue lines represent the 99% CI for the OS curve. Black circles at each time point of interest represent the UPL, MLV, and LPL. The black lines between the time points of interest connect the MLVs and are not indicative of a formal extrapolation method. Abbreviations: CI, confidence interval; LPL, lower plausible limit; MLV, most likely value; OS, overall survival; UPL, upper plausible limit

## Consensus meeting (group-level elicitation)

The consensus meeting involved: 1) reviewing anonymized estimates from the individual interviews; 2) identifying points of divergence among the individual estimates and discussing alternative rationales; and 3) eliciting group consensus estimates for the UPLs, LPLs, and MLVs for survival at 3, 5, and 10 years for each study of interest. Experts were anonymized during the teleconference (e.g. Expert 1) to reduce the potential for bias, such as dominance or deferment based on perceptions of fellow experts.

In accordance with the SHELF methodology, experts were asked to adopt the viewpoint of a ‘rational impartial observer’ when reaching group consensus [23], which represented an external observer (i.e. an objective decision-maker) who had observed the results from the individual estimates, had listened to the group discussion, and understood all arguments. It was explained that it was not expected for experts to reach complete agreement, or for the consensus values to directly represent their individual opinions, but rather, experts were reminded to consider discussion points, objectively weight the different insights, and reach consensus on what a rational impartial observer would believe. The experts were asked to not select values to simply satisfy or include all opinions from all experts.

Following discussions, the same web-based application from the individual interviews was used to elicit and visualize the group consensus values, where experts were asked to confirm they agreed regarding the distribution of the most likely values within the plausible limits at each time point, as well as the clinical plausibility of the shape of curves based on all consensus values. If any expert was dissatisfied, then the basis for their concern was discussed and an opportunity to revise the estimates was provided.

Prior to eliciting group consensus values, experts identified that more optimistic values from the individual interviews may have been driven by beliefs that a small subset of patients may experience long-term remission and thus survive. In contrast, other experts noted that they adopted a more pessimistic view due to the belief that not many patients would have prolonged remissions given that the patients were heavily pretreated, highly refractory, and potentially had high-risk cytogenetics, and were therefore unlikely to survive long term. The experts also noted variation in estimates at early time points due to different clinical experiences and observations regarding the number of patients that survive beyond 3 years, whereas there was a common belief that it was unlikely for patients to be alive at 10 years.

Experts agreed that long-term survival may be related to both treatment and non-aggressive disease features. A small proportion of patients may experience disease relapse without the disease becoming more aggressive in terms of molecular basis or extramedullary disease. These patients may respond to treatments and again experience prolonged remission and survival. More broadly, experts expressed those patients receiving chimeric antigen receptor (CAR) T cell therapy in the KarMMa trial were already at an aggressive disease phase so were experiencing disease relapse consistently, explaining why benefits from treatment with ide-cel may not lead to long-term remission.
