# Supplementary material for: Structured expert elicitation to inform long-term survival extrapolations using alternative parametric distributions: a case study of CAR T therapy for relapsed/ refractory multiple myeloma
Source: BMC Med Res Methodol. 2022 Oct 15;22:272. doi: 10.1186/s12874-022-01745-z (PMC9569052; doi:10.1186/s12874-022-01745-z)
Supplement: Supplementary file 2 — Additional file 2. Parameterizations of alternative models. Table 2. Parameterizations of survival distributions employed in analyses. [file 12874_2022_1745_MOESM2_ESM.docx]

# Additional File 2. Parameterizations of alternative models

Table 2 Parameterizations of survival distributions employed in analyses

| Distribution | Probability density function | Parameters |
| --- | --- | --- |
| Exponential | λe^−λt^ | Rate = λ>0 |
| Weibull | vλt^v−1^ exp(−λt^v^ ) | Shape = v>0  Rate = λ>0 |
| Gompertz | α exp(−βt) exp{−α β [1 − exp(−βt)]} | Shape = α > 0  Scale = β ∈ (−∞,∞) |
| Gamma | λ^r^t^r−1^ exp(−λt) / Γ(r) | Shape = r > 0  Scale = λ > 0 |
| Lognormal | $\left( \frac{\tau}{2\pi} \right)^{\frac{1}{2}}t^{-1}exp\{-\frac{\tau\left( \log\left( t \right)-\mu\right)^{2}}{2}\}$ | Mean log = μ ∈ (−∞,∞)  Shape = τ>0 |
| Log-logistic | (ρλ) (λt) ^ρ-1^ / (1 + (λt) ^ρ^) ^2^ | Shape=ρ>0  Scale=λ>0 |
| Generalized gamma | bλ^br^t^br−1^ exp{−(λt) b} / Γ(r) | r > 0, b > 0, λ > 0 |
