# Supplementary material for: Structured expert elicitation to inform long-term survival extrapolations using alternative parametric distributions: a case study of CAR T therapy for relapsed/ refractory multiple myeloma
Source: BMC Med Res Methodol. 2022 Oct 15;22:272. doi: 10.1186/s12874-022-01745-z (PMC9569052; doi:10.1186/s12874-022-01745-z)
Supplement: Supplementary file 4 — Additional file 4: Fig. 3. Long-term survival estimates and 95% CrIs based on observed KarMMa data (without expert opinion). Fig. 4. Long-term survival estimates and 95% CrIs based on observed KarMMa data and consensus expert opinion. [file 12874_2022_1745_MOESM4_ESM.docx]

# Additional File 4.

**Fig. 3** Long-term survival estimates and 95% CrIs based on observed KarMMa data (without expert opinion)


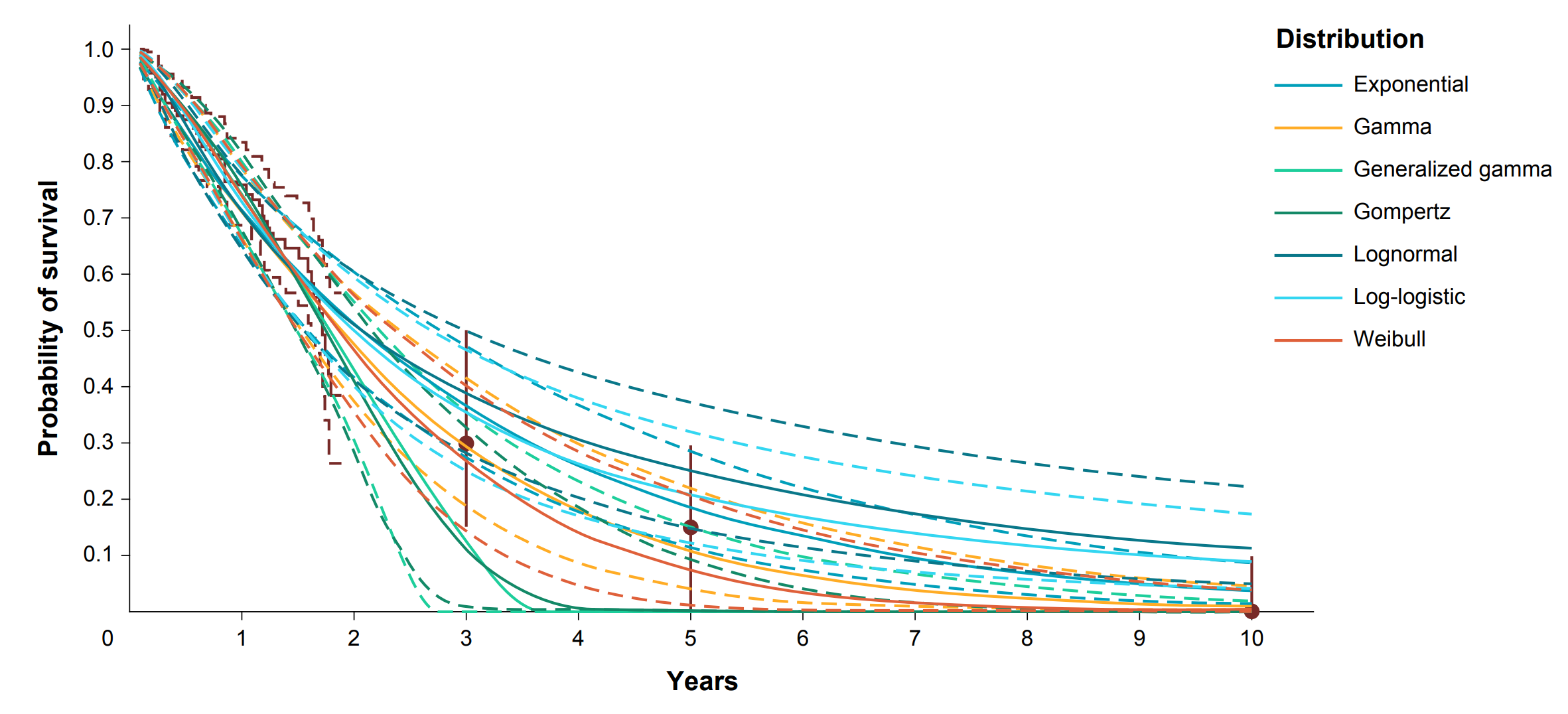


Observed data includes OS curve (solid line) and associated 99% CI (dashed lines). Dots represent consensus most likely values, and vertical bars show the plausible range. Abbreviations: CI, confidence interval; OS, overall survival

**Fig. 4** Long-term survival estimates and 95% CrIs based on observed KarMMa data and consensus expert opinion


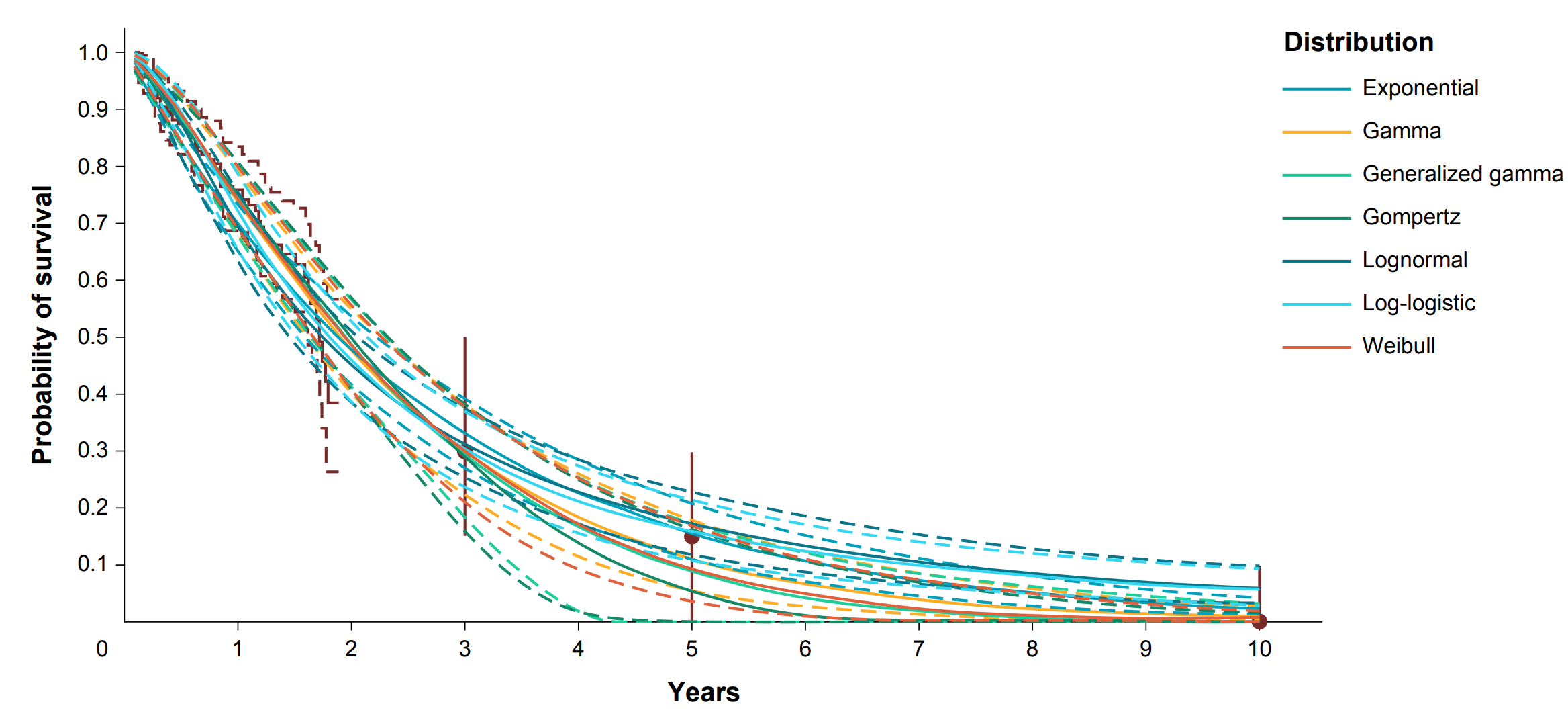


Observed data includes OS curve (solid line) and associated 99% CI (dashed lines). Dots represent consensus most likely values, and vertical bars show the plausible range. Abbreviations: CI, confidence interval; OS, overall survival
